# Reconsidering the Active Psychological Ingredients Underlying Intercultural Adaptation: Implications for International Business

**DOI:** 10.3389/fpsyg.2020.529737

**Published:** 2020-09-24

**Authors:** David Matsumoto, Hyisung C. Hwang

**Affiliations:** ^1^Psychology Department, San Francisco State University, San Francisco, CA, United States; ^2^Humintell, El Cerrito, CA, United States

**Keywords:** intercultural adaptation, intercultural adjustment, culture-general competence, MPQ, CQS, ICAPS

## Abstract

A major issue facing many businesses today, both large and small, concerns intercultural adaptation, and more broadly, diversity. Many businesses struggle with their employees sent to different countries and cultures to adapt effectively in host cultures, as well as for their home culture employees to adapt effectively to changing environments brought on by visitors from other cultures and other sources of diversity. To address this issue, many tests and measures have been developed to identify the core psychological skills, competencies, and aptitudes underlying intercultural adaptation. Elucidation of such skills and competencies would have multiple theoretical and practical ramifications. A recent review of this literature indicated that three tests – the Multicultural Personality Questionnaire, the Cultural Intelligence Scale, and the Intercultural Adjustment Potential Scale – had the best psychometric evidence for validity to date. No study, however, has examined the statistical overlap among these tests; which scales or combination of scales best predict adaptation; and most importantly, what are possible, yet unassessed, constructs underlying them. The purpose of this study was to examine these three questions initially. Non-immigrant, non-sojourner convenience samples from four countries/language groups completed all three tests and a measure of life satisfaction as a proxy for adaptation. Scales from the three tests were moderately – highly intercorrelated and predicted adaptation. A combination of scales from the tests best predicted adaptation, better than scales from any one test. Analyses examining the latent structures underlying the combined tests suggested several psychological constructs new to the intercultural adaptation literature. We discuss the implications of these findings for theory and application in international business.

## Introduction

The last half century has witnessed sociocultural changes in many countries and cultures in economic prosperity and affluence, for better and worse. Borders have become increasingly porous, and communication and transportation technologies have brought people closer than ever before. These changes have been felt in many societies as they manage evolving issues concerning sojourning, migration, and immigration, all topics at the forefront of everyday life.

International businesses have not been immune from these changing sociocultural landscapes. Many companies send employees (and families) to different countries and cultures for short to extended periods and receive workers from other countries and cultures. As societies become increasingly diverse, with cultural, economic, ethnic, gender, and generational differences all contributing factors, businesses also deal with very diverse workforces and customer bases.

Correspondingly, research and theory on culture and organizations have evolved. Seminal works on culture shock (Oberg, 1960; Furnham and Bochner, 1982; Pederson, 1995; Ward et al., 2001), reverse culture shock (culture shock that individuals experience when returning to their home cultures after living in a different culture for an extended period of time; Gaw, 2000; Yoshida et al., 2002), and negotiation issues concerning Japanese auto manufacturers in the United States (Graham, 1993) have opened the doors to new and exciting research and theory relevant to international and intercultural business (Trompenaars and Hampden-Turner, 2012). Studies and theories now reflect the fact that businesses today from all countries and continents are in all countries and continents, crisscrossing the globe in a complex international and intercultural web.

### Intercultural Adaptation and a Search for Its Core and Psychological Components

For businesses, increasing cultural diversity comes with increased risk, as diverse, intercultural workgroups are often associated with greater conflict, stress, turnover, and absenteeism, and less trust, cohesion, and job satisfaction (Ayoko et al., 2004). These risks, as well as macrolevel sociocultural changes, bring the topic of intercultural adaptation to the forefront. For businesses, a contributor to organizational success revolves around how organizations adapt and evolve to ever-changing environmental and contextual landscapes to keep up with sociocultural changes of the last half century. Businesses are comprised of individuals, and business-to-business and business-to-customer interactions are essentially interactions among individuals, and at its elemental level, individual-level intercultural adaptation becomes crucial for effective performance both home and abroad.

Intercultural adaptation has been a major topic of research and theory in mainstream academia and has been studied from a variety of viewpoints such as acculturation (Berry and Sam, 1997; Berry(ed.), 2017), sociocultural adaptation (Searle and Ward, 1990; Ward and Szabo, 2019), biculturalism (Nguyen and Benet-Martínez, 2013), and cultural distance (Furnham and Bochner, 1982; Geeraert and Demoulin, 2013; Demes and Geeraert, 2014). One segment of this literature has focused on identifying the active psychological ingredients underlying intercultural adaptation – skills, competencies, aptitudes, and attitudes – that would facilitate constructive adaptation. These attempts are worthwhile as they have highlighted a possible core psychological engine necessary for successful adaptation in a culture-general approach (Brislin, 1986; Cushner, 1989; Bhawuk and Brislin, 2000).

Within this genre, there have been many attempts to develop and validate tests of such psychological components over the years. A review of the psychometric properties of ten such tests (Matsumoto and Hwang, 2013) indicated that three had the best validity and reliability evidence available – the Multicultural Personality Questionnaire (MPQ), the Cultural Intelligence Scale (CQS), and the Intercultural Adjustment Potential Scale (ICAPS). The MPQ is a 91-item personality test designed to assess behavior when interacting with people from different cultures and to predict how well how people adapt to other cultures (van der Zee and Van Oudenhoven, 2000, 2001). It includes five scales: Cultural Empathy – the capacity to identify with thoughts, feelings, and behavior of people from different cultures; Openmindedness – the ability to be open and unprejudiced when encountering people from different cultures; Social Initiative – the tendency to approach social situations actively and with initiative; Emotional Stability – the ability to remain calm in stressful situations; and Flexibility – the ability to adjust one’s behavior to new and unknown situations.

The CQS is a 20-item test that assesses an individual’s capability to manage and function effectively in culturally diverse settings (Ang et al., 2006, 2007). It contains four scales: Metacognitive CQ – consciousness and awareness during intercultural interactions; Cognitive CQ – cultural knowledge of norms, customs, practices, and conventions; Motivational CQ – ability to direct attention and energy toward cultural differences; and Behavioral CQ – the capability to exhibit appropriate verbal and nonverbal behavior in intercultural interactions.

The ICAPS is a 55-item test that assesses individual strengths and weaknesses in being able to adapt interculturally (Matsumoto et al., 2001, 2003, 2004). It produces five scores: Overall Adjustment Potential – one’s overall potential to adjust well interculturally; Emotional Robustness – the ability to monitor and manage emotional experiences and expressions and to channel them in constructive ways; Openness – the desire for new experiences, emotions, and thoughts; Flexibility – the ability to assimilate new experiences, schema, and ways of thinking; and Critical Thinking – the ability to think outside the box in creative and autonomous ways.

### Searching for Conceptual Clarity About What Is Measured

A question raised by the previous review and others (Matsumoto and Hwang, 2013; Leung et al., 2014) concerned conceptual ambiguity about these tests and scales (and other similarly constructed measures in this genre) and what exactly is being measured. Collectively, the tests purportedly assess personality traits (MPQ), capabilities (CQS), and a combination of traits and capabilities (ICAPS). The MPQ, for instance, assesses traits that predispose individuals’ sensitivities to threats or challenges which, in turn, buffer stress from culture shock and facilitate cultural learning (van der Zee and van Oudenhoven, 2013). The CQS was based on “contemporary theories of intelligence” (Sternberg and Detterman, 1986). Although one of its scales – Cognitive CQS – assesses cultural knowledge, the other three assess abilities and actual behavior (Metacognitive, Motivational, and Behavioral CQS). Likewise, the ICAPS scales assess a combination of traits (e.g., Openness, Emotional Robustness) and abilities/skills (Flexibility, Critical Thinking).

Despite what each test purports to measure, they all use a common methodology – self-report – and because of that, questions exist concerning what is really being assessed (for extended discussion see Baumeister et al., 2007), especially concerning abilities, capabilities, skills, and behavior. There is ample evidence that self-reports of abilities do not necessarily correlate with actual abilities (Little et al., 1995, 2003; Baumeister et al., 2003; Rodriguez et al., 2003; Pyszcynski et al., 2004) and that self-reports of behavior often do not always reflect actual behavior (West and Brown, 1975; Nisbett and Wilson, 1977; Hessing et al., 1988; Barr and Kleck, 1995; Woodzicka and LaFrance, 2001, 2005; Wilson and Gilbert, 2003; Matsumoto, 2006; Baumeister et al., 2007; Woodzicka, 2008; Kawakami et al., 2009). These reviews and studies raise conceptual questions concerning what actually might be measured in scales like Metacognitive, Motivational, and Behavioral CQS, and ICAPS Flexibility and Critical Thinking. Additional conceptual questions also exist, such as whether individuals can self-report about meta-consciousness and awareness.

This conceptual ambiguity about what is being measured is compounded by reports of each test predicting intercultural adaptation over and above standard Big Five personality measures (e.g., Matsumoto et al., 2007; Rockstuhl and Van Dyne, 2018). Although such findings have typically been interpreted as the target test assessing something other than personality, there is a possibility that the tests, in part, were derived conceptually and empirically (e.g., using factor analytic techniques) to maximize prediction of intercultural adaptation by capturing unique elements of personality that are optimal for intercultural success and thus themselves assess personality. That is, although the scales of the various tests have been derived from slightly different traditions, they seem to display conceptual overlap.

One way to address initially these conceptual questions is to measure all three tests together in people from different cultures, merge the data in a pancultural analysis (Leung and Bond, 1989), and identify the constellation of core constructs underlying them. The generated constructs may be similar to those currently purported by each test. However, different constructs may also emerge, and the current constructs may exist because of their association with these underlying, yet unassessed, constructs (Ward et al., 2009, Study 3 did use two tests – the MPQ and CQS – and reported moderate correlations among their scales, but an analysis merging the two tests was not conducted).

Identifying such constructs would be a boon for the field, as they may provide theoretical clarity about the core, psychological components of intercultural adaptation. At the very least, they would provide an alternative viewpoint to conceptualizing what those components may be. Conceptual clarity would fuel further empirical work and bring more precision to applied work in intercultural training. We examine these possibilities in this study.

### How Do the Tests Predict Adaptation Relative to Each Other?

A secondary question raised by the previous review (Matsumoto and Hwang, 2013) concerned the degree to which the three tests predicted adaptation relative to each other (the answer to which also has implications to the conceptual issue described above). One way to examine this question is to assess them together in the same study along with criterion variables assessing adaptation. One study did so using two tests (Ward et al., 2009, Study 3). International students completed the MPQ and CQS along with four scales assessing adaptation – the Satisfaction with Life Scale (SWLS; Diener et al., 1985; Pavot and Diener, 2013), the Zung Self-Rating Depression Scale (Zung, 1965), the Sociocultural Adaptation Scale (SCAS; Ward and Kennedy, 1999), and an Academic Adaptation Scale, a domain-specific version of the SCAS focusing on academic difficulties. The CQS scales did not significantly predict any of the adaptation variables beyond the MPQ and demographics; MPQ Emotional stability was the only significant predictor in two analyses.

Although those findings were revealing, analyses merging the two tests were not reported. Such an analysis would have allowed for a search for higher-order latent constructs (related to the above discussion concerning conceptual clarity) and for predictors of intercultural adaptation that resulted from a combination of tests and that may not have been apparent from the scales scored. The non-findings for incremental validity of one of those tests (CQS) to predict adaptation over another (MPQ) raised further questions concerning their conceptual overlap. Addressing this issue is especially important in considering the elemental psychological ingredients that predict intercultural adaptation for laypersons and businesses alike, as questions concerning such components have been increasingly important in the business world (Ayoko and Hartel, 2002; Ayoko et al., 2004).

The goal of such a comparison is not to see which is better or best. Instead, related to the conceptual discussion above, there is a possibility that each test measures something different than previously thought and that they may differentially predict adaptation. If so, then a combination of their scales would maximally predict adaptation, something that would not be witnessed when examining each test singly. This study examined these possibilities by including a measure of adaptation to test these ideas initially.

### Overview and Hypotheses

We addressed the goals above by assessing the three tests discussed above along with a measure of adaptation across different countries/language groups. The purpose of this study was to examine the statistical overlap among the tests; which scales or combination of scales best predicted adaptation; and most importantly, search for possible underlying, yet unassessed, constructs of the tests. Answers to these questions would have implications not only for theoretical perspectives related to intercultural adaptation but also to practical applications in businesses and other settings. We predicted that (1) a combination of scales across the tests would best predict adaptation, and (2) psychological constructs based on a superordinate merging of the tests would align differently than those of the original scales.

## Materials and Methods

### Participants

Participants included a convenience sample of *N* = 353 individuals from four countries (language groups): United States (English), *n* = 112, 65% female, *M*_*age*_ = 30.85, *SD*_*age*_ = 12.36; People’s Republic of China (Chinese), *n* = 173, 81% female, *M_age_* = 22.96, *SD*_*age*_ = 8.52; South Korea (Korean), *n* = 35, 74% female, *M*_*age*_ = 26.06, *SD*_*age*_ = 11.39; and France (French), *n* = 33, 44% female, *M*_*age*_ = 24.13, *SD*_*age*_ = 13.26. (An initial sample of *N* = 489 was collected, but cases were excluded when more than 10% of data for a case were missing.) All reported being born and raised in their respective country and as being fluent in reading, writing, and speaking the respective language. Approximately half the United States and Chinese samples were recruited using an online survey platform; the other half of both groups and participants in South Korea and France were recruited by local assistants in the San Francisco Bay area, CA; Beijing, China; Seoul, South Korea; and Bordeaux, France. Participants were recruited for a different study (Matsumoto and Hwang, 2015) and opted to complete the procedures here as an additional study after the initial study was completed.

### Tests

The MPQ is a 91-item test with five-point scales labeled 1, Totally not Applicable; 2, Hardly Applicable; 3, Moderately Applicable; 4, Largely Applicable; and 5, Completely Applicable (van der Zee and Van Oudenhoven, 2000, 2001). Five scales are computed after reverse coding certain items (sample items included in quotes): Cultural Empathy (18 items; “Understands other people’s feelings,” “Tries to understand other people’s behavior”), Openmindedness (18 items; “Is interested in other cultures,” “Is fascinated by other people’s opinions”), Social Initiative (17 items; “Takes initiatives,” “Makes contacts easily”), Emotional Stability (20 items; “Is not easily hurt,” “Keeps calm at ill-luck”), and Flexibility (18 items; “Likes low-comfort holidays,” “Changes easily from one activity to another”).

The CQS is a 20-item test with seven-point scales labeled 1, Strongly Disagree and 7, Strongly Agree (Ang et al., 2006, 2007). Four scales are computed: Metacognitive CQ (four items; “I adjust my cultural knowledge as I interact with people from a culture that is unfamiliar to me,” “I am conscious of the cultural knowledge I apply to cross-cultural interactions”), Cognitive CQ (six items; “I know the marriage systems of other cultures,” “I know the arts and crafts of other cultures”), Motivational CQ (five items; “I enjoy interacting with people from different cultures,” “I enjoy living in cultures that are unfamiliar to me”), and Behavioral CQ (five items; “I vary the rate of my speaking when a cross-cultural situation requires it,” “I change my non-verbal behavior when a cross-cultural situation requires it”).

The ICAPS is a 55-item test with seven-point scales labeled 1, Strongly Disagree and 7, Strongly Agree (Matsumoto et al., 2001, 2003, 2004). Five scales are computed after reverse coding certain items: Overall Adjustment Potential (55 items), Emotional Robustness (16 items; “I rarely feel anxious or fearful,” “I am usually good at dealing with emergencies”), Openness (15 items; “Only a stupid person would try to change our traditional way of life,” “It is better to not trust anyone”), Flexibility (15 items; “I have tried to write poetry,” “Smells remind me of old memories”), and Critical Thinking (nine items; “Some people are just no good,” “Everyone should follow rules”). Scores were standardized to means of 50 and SD 10.

We utilized the Satisfaction with Life Scale (SWLS; Diener et al., 1985; Pavot and Diener, 2013) as a measure of general adaptation. It contains five items (e.g., “I am satisfied with my life”) using seven-point scales labeled 1, Strongly Disagree; 2, Disagree; 3, Slightly Disagree; 4, Neither Agree nor Disagree; 5, Slightly Agree; 6, Agree; and 7, Strongly Agree. A single score is computed by averaging the scores. Readers are cautioned that, because the samples were not immigrants or sojourners, scores on the SWLS did not indicate intercultural adaptation but instead general adaptation; thus, interpretations of findings involving the SWLS should be tempered accordingly. This limitation, however, did not detract from the main purpose of the study, which was merging the three target tests above and a combined analysis of them by themselves.

We also administered the Self-Monitoring Scale (SMS; Snyder, 1974) as a covariate. It assesses the degree to which individuals will modify how they are perceived by others and contains 25 true/false items (e.g., “I find it hard to imitate the behavior of other people”). A single score is computed by counting true or false responses across the 25 items.

Although all tests used in this study have been demonstrated to have construct and criterion validity across many countries and cultures (van der Zee and Van Oudenhoven, 2000, 2001; Matsumoto et al., 2001, 2003, 2004; Ang et al., 2006, 2007), to establish cross-cultural structural equivalence in this study, we computed Exploratory Factor Analyses (EFAs) on the MPQ, CQS, ICAPS, and SWLS separately for the two largest samples, United States and China. Separate EFAs by group are preferable than Confirmatory Factor Analyses to establish structural validity of a test (van de Vijver and Poortinga, 2002; van de Vijver and Leung, 2011). SWLS converged in a single factor, and CQS converged on four factors across countries using both Kaiser criterion and scree plot; MPQ and ICAPS converged on five and four factors, respectively, using scree plot. These results provided some justification for the structural equivalence for all tests commensurate with the recommended scoring procedures, in two culturally divergent samples. Cross-cultural equivalence in reliabilities was demonstrated in analyses below (Table 1).

**TABLE 1:** Means, SD, and alphas for all scales separately for each country/language group.

			Chinese	English	French	Korean	Total
MPQ	Cultural empathy	M	3.63	3.66	3.77	3.67	3.65
		SD	0.43	0.62	0.38	0.42	0.47
		α	0.81	0.90	0.82	0.79	0.85
	Openmindedness	M	3.40	3.47	3.29	3.51	3.42
		SD	0.46	0.59	0.37	0.49	0.49
		α	0.81	0.89	0.75	0.83	0.83
	Social initiative	M	3.12	3.20	3.49	3.39	3.18
		SD	0.45	0.57	0.47	0.45	0.49
		α	0.79	0.87	0.84	0.76	0.81
	Emotional stability	M	3.00	3.02	3.08	3.08	3.02
		SD	0.39	0.55	0.50	0.52	0.45
		α	0.75	0.84	0.87	0.82	0.79
	Flexibility	M	2.91	2.93	3.10	3.37	2.97
		SD	0.32	0.46	0.36	0.39	0.39
		α	0.55	0.77	0.74	0.68	0.68
CQS	Metacognitive	M	4.36	5.00	5.18	5.33	4.64
		SD	1.06	1.20	0.99	0.96	1.14
		α	0.72	0.92	0.83	0.69	0.46
	Cognitive	M	3.34	3.94	3.50	4.37	3.57
		SD	1.28	1.38	1.06	1.26	1.33
		α	0.92	0.92	0.80	0.89	0.75
	Motivational	M	4.38	4.83	4.50	5.34	4.57
		SD	1.20	1.24	0.89	1.18	1.22
		α	0.84	0.91	0.73	0.88	0.76
	Behavioral	M	5.15	4.78	4.40	5.19	5.02
		SD	1.14	1.26	1.27	1.55	1.23
		α	0.89	0.90	0.83	0.95	0.51
ICAPS	Overall adjustment potential	M	46.72	49.96	51.95	46.94	47.77
		SD	9.76	9.20	7.42	9.69	9.61
		α	0.50	0.41	0.26	0.46	0.41
	Emotional robustness	M	47.73	50.44	58.84	49.70	49.25
		SD	8.70	9.64	8.78	10.47	9.48
		α	0.72	0.75	0.79	0.81	0.79
	Openness	M	48.69	47.38	52.08	54.64	49.20
		SD	8.73	10.41	7.30	7.90	9.15
		α	0.74	0.82	0.65	0.69	0.91
	Flexibility	M	49.67	54.09	48.37	47.23	50.29
		SD	7.57	8.27	5.60	9.22	8.02
		α	0.47	0.59	0.20	0.62	0.87
	Critical thinking	M	46.48	47.73	45.95	42.77	46.37
		SD	7.89	10.16	6.56	7.74	8.39
		α	0.40	0.67	0.10	0.26	0.89
SWLS	SWLS	M	17.40	23.66	26.35	23.45	19.90
		SD	5.54	7.07	3.20	7.97	6.86
		α	0.75	0.92	0.60	0.94	0.86
SMS	Self-monitoring scale	M	11.36	12.23	11.61	13.35	11.74
		SD	3.67	4.05	3.94	4.06	3.84
		α	0.61	0.67	0.70	0.72	0.63

### Procedures

All procedures were administered online, and all protocols were translated into the target languages by a professional translation company that verified accuracy of the translations through back-translation and/or committee approach. Local assistants also confirmed the readability and understandability of the protocols. Respondents first provided basic demographic data concerning sex, age, ethnicity, places of birth and upbringing, first and other languages, and confirmation of fluency in reading, writing, and speaking the target language. They then completed the SMS and SWLS; the MPQ, CQS, and ICAPS followed in a counterbalanced order.

## Results

### Preliminary Analyses

We computed descriptive statistics (means and SDs) and Cronbach’s alphas on all scales separately for each of the language groups (Table 1). All data appeared to be within normal ranges. Low alphas for the ICAPS OAP, Flexibility, and Critical Thinking scales have been reported since the development and initial validation of the scale (Matsumoto et al., 2001, 2003) and as explained previously (Matsumoto and Hwang, 2013) were artifacts of the item derivation and scale validation procedures.

One-way ANOVAs on each of the scales comparing all groups and the English and Chinese only produced many statistically significant effects (11/14 tests for the entire sample, 7/14 for English-Chinese only). We standardized each of the scale scores within each group to conduct a pancultural analysis, which eliminated cultural differences and possible positioning effects of those differences in all regression-based analyses (Leung and Bond, 1989). Subsequent analyses were thus based on the entire sample, consistent with a culture-general analysis.

### Associations Among the Scales

Intercorrelations among the standardized scales indicated consistent low–moderately sized associations among them (Table 2). We also computed canonical correlations among the three pairs of tests; all were highly correlated, *CC* = 0.72, *λ* = 0.38, *F*(20, 1105.39) = 18.75, *p* < 0.001; *CC* = 0.77, *λ* = 0.21, *F*(25, 1227.40) = 25.83, *p* < 0.001; and *CC* = 0.54, *λ* = 0.58, *F*(20, 1098.75) = 9.84, *p* < 0.001, for MPQ and CQS, MPQ and ICAPS, and CQS and ICAPS, respectively. (For the ICAPS, the OAP score was not included in any multivariate analysis reported here or below.) These findings indicated substantial degrees of overlap among the three tests but also some degree of uniqueness.

**TABLE 2:** Intercorrelations among scales and age.

	MPQ				CQS				ICAPS					SWLS	SMS	Age
	OP	SI	ES	FL	ME	CO	MO	BE	OAP	ER	OP	FL	NA			
**MPQ**																
CE	0.61**	0.42**	0.11*	0.01	0.42**	0.17**	0.41**	0.36**	0.34**	0.17**	0.13*	0.41**	0.38**	0.14*	0.12*	0.02
OP		0.51**	0.20**	0.12*	0.53**	0.48**	0.65**	0.38**	0.36**	0.15**	–0.08	0.56**	0.29**	0.16**	0.16*	0.12*
SI			0.40**	0.33**	0.36**	0.41**	0.47**	0.22**	0.44**	0.45**	0.13*	0.25**	0.11*	0.31**	0.30**	0.10
ES				0.30*	0.16**	0.25**	0.27**	0.11*	0.53**	0.72**	0.16**	0.05	–0.05	0.33**	0.02	0.11*
FL					–0.01	0.16**	0.25**	−0.06	0.22**	0.30**	0.30**	–0.01	−0.33**	0.11*	0.20**	–0.12
**CQS**																
ME						0.51**	0.53**	0.33**	0.36**	0.23**	0.02	0.40**	0.23**	0.29**	0.15**	−0.26**
CO							0.49**	0.18**	0.20**	0.27**	−0.12*	0.22**	0.03	0.27**	0.11*	0.17**
MO								0.44**	0.35**	0.23**	0.03	0.37**	0.13*	0.18*	0.27**	0.06
BE									0.19**	–0.05	–0.02	0.30**	0.30**	−0.13*	0.17**	0.03
**ICAPS**																
OAP										0.67**	0.38**	0.53**	0.12*	0.30**	0.04	0.20**
ER											0.31**	0.05	–0.10	0.42**	–0.04	0.18**
OP												−0.11*	−0.31**	0.06	–0.09	0.02
FL													0.32**	0.09	0.19**	0.09
CT														0.10	0.02	0.21**
SWLS															0.07	0.18**
SMS																−0.16**

*p < 0.05, **p < 0.01. MPQ CE, Cultural Empathy; MPQ OP, Openmindedness; MPQ SI, Social Initiative; MPQ ES, Emotional Stability; MPQ FL, Flexibility; CQS ME, Metacognitive; CQS CO, Cognitive; CQS MO, Motivational; CQS BE, Behavioral; ICAPS OAP, Overall Adjustment Potential; ICAPS ER, Emotional Robustness; ICAPS OP, Openness; ICAPS FL, Flexibility; ICAPS CT, Critical Thinking; SWLS, Satisfaction with Life Scale; SMS, Self-Monitoring Scale.

### Hierarchical Regressions Predicting SWLS

#### Separately for Each Test

We examined how well each test predicted SWLS by computing simultaneous multiple regressions (Table 3). Because of their bivariate associations (Table 2), age and SMS were included in the first step of all regression analyses but did not produce any significant findings and are therefore not mentioned further. All tests produced significant overall models. For MPQ, Social Initiative, Emotional Stability, and Flexibility contributed independently to the prediction of SWLS. For CQS, the Cognitive and Behavioral scales contributed independently to the prediction. For ICAPS, Emotional Robustness and Critical Thinking contributed independently. However, there were some unexpected directional findings: MPQ Flexibility and CQS Behavioral were negatively related with SWLS. The negative association with CQS Behavioral was consistent with meta-analysis findings in Rockstuhl and Van Dyne (2018). These counterintuitive findings raised questions concerning the interpretation of these scales when their associations with other scales on the same test were accounted for.

**TABLE 3:** Results of multiple regressions predicting SWLS.

Predictors	*R*	*F*	df_1_	df_2_	*p*	Variable In	*β*	RW	LLCI	ULCI	RS-RW
MPQ	0.37	10.70	5	336	<0.001	MPQ CE	–0.01	0.007	0.002	0.028	4.69
						MPQ OP	0.04	0.007	0.002	0.021	4.83
						MPQ SI	0.14*	0.054*	0.019	0.103	37.13
						MPQ ES	0.30**	0.074*	0.028	0.136	50.64
						MPQ FL	−0.12*	0.004	0.001	0.012	2.71
CQS	0.26	6.21	4	337	<0.001	CQS ME	0.12	0.062*	0.024	0.113	36.29
						CQS CO	0.16*	0.042*	0.013	0.084	24.33
						CQS MO	0.06	0.022*	0.008	0.051	13.00
						CQS BE	−0.19*	0.045*	0.014	0.095	26.39
ICAPS	0.38	13.91	4	337	<0.001	ICAPS ER	0.37**	0.179*	0.108	0.259	89.79
						ICAPS OP	–0.05	0.005	0.001	0.009	2.40
						ICAPS FL	0.01	0.004	0.000	0.023	2.19
						ICAPS CT	0.12*	0.112	0.001	0.040	5.61
All scales from three tests	0.43	15.46	5	336	<0.001	ICAPS ER	0.19*	0.107*	0.061	0.158	40.76
						ICAPS CT	0.19**	0.020	0.003	0.054	7.74
						CQS BE	−0.18*	0.031	0.007	0.070	11.80
						CQS CO	0.14*	0.052*	0.018	0.099	19.75
						MPQ ES	0.18*	0.052*	0.023	0.092	19.94
Component scores	0.33	13.83	3	338	<0.001	Component 1	0.01	0.005	0.001	0.027	5.87
						Component 2	0.31**	0.073*	0.027	0.136	83.91
						Component 3	0.11*	0.009	0.000	0.035	10.22
New scale scores	0.43	15.17	5	336	< 0.001	Assertiveness	0.19*	0.041*	0.014	0.083	22.15
						Attentiveness to Others	0.18*	0.020*	0.005	0.056	10.96
						Emotional Robustness	−0.35**	0.105*	0.046	0.174	56.88
						Caution	0.09	0.003	0.001	0.012	2.06
						Self-Awareness	−0.20*	0.015*	0.004	0.041	7.95

*^∗^*p* < 0.05. ^∗∗^*p* < 0.001. MPQ CE, Cultural Empathy; MPQ OP, Openmindedness; MPQ SI, Social Initiative; MPQ ES, Emotional Stability; MPQ FL, Flexibility; CQS ME, Metacognitive; CQS CO, Cognitive; CQS MO, Motivational; CQS BE, Behavioral; ICAPS OAP, Overall Adjustment Potential; ICAPS ER, Emotional Robustness; ICAPS OP, Openness; ICAPS FL, Flexibility; ICAPS CT, Critical Thinking; RW, Raw Relative Weight; LLCI, lower level confidence interval used to test the significance of RW; ULCI, upper level confidence interval used to test the significance of RW; RS-RW, RW rescaled as a percentage of predicted variance in SWLS attributed to each predictor.*

Because results from regressions may overfit data, and because direct comparisons of standardized regression coefficients to determine relative contributions of each predictor do not take into account multicollinearity among the predictors, we also computed relative weights analyses (RWA) for all regression results (Johnson, 2000; Tonidandel and LeBreton, 2015) as well as their statistical significance (Tonidandel et al., 2009). RWA involves variable transformations that produce statistics for each predictor that estimate the relative importance of each predictor orthogonally to the other predictors. RWA produces a number of statistics for each predictor variable; we report four: the Raw Relative Weight (RW), the proportion of variance in the outcome variable attributed to that predictor; the lower (LLCI) and upper level confidence intervals (ULCI) used to test the significance of RW against a randomly generated predictor variable with RW = zero; and RW rescaled as a percentage of predicted variance in the outcome variable attributed to each predictor relative to the total amount of variance attributed to all predictors (not total variance in the outcome variable; RS-RW).

For the MPQ, MPQ Social Initiative and Emotional Stability both contributed significantly to the prediction of SWLS, accounting for 37.13 and 50.64% of the prediction, respectively. For the CQS, all four scales contributed significantly to the prediction of SWLS, with the largest contribution from CQS Metacognitive. For the ICAPS, Emotion Regulation and Critical Thinking contributed significantly to the prediction, with Emotion Regulation contributing almost 90% to the prediction.

We also tested for differences between the United States and Chinese data, the two largest samples, on all RWA above, and arguably the most culturally divergent. None of the RWA findings differed between these two groups.

#### Using Combined Scales

To examine how the tests combined could predict SWLS, we computed stepwise multiple regressions on SWLS including all three tests’ scales. The analysis produced five models; the final model indicated that ICAPS Emotional Robustness and Critical Thinking, CQS Behavioral and Cognitive, and MPQ Emotional Stability contributed independently to the prediction (Table 3). As above the coefficient for CQS Behavioral was negative, indicating that lower scores on this scale were associated with greater SWLS. All other scales in the final model were positively associated with SWLS. RWA indicated that three scales contributed significantly to the prediction of SWLS. ICAPS Emotion Regulation accounted for almost 41% of the prediction, while CQS Cognitive and MPQ Emotional Stability each accounted for almost 20% of the prediction. There were no differences in RWA analyses between the United States and Chinese samples.

Thus, a combination of scales from the three tests was optimal in predicting adjustment, as predicted. The final model produced an *R* = 0.43, which accounted for a larger amount of variance in SWLS than any of the scales separately. This finding could not have been accounted for simply by the use of more predictors because the final model included five variables, the same number of scales as the MPQ. Model 4, which included four predictors, included the same number of scales as the CQS or ICAPS and had a larger effect on SWLS (*R* = 0.42) than either of these scales separately. We concluded that the scales of the various tests measured something unique vis-à-vis their ability to predict adaptation; thus, subsequent analyses examined the existence of factors superordinate to the three tests.

### Search for Empirical Evidence of Superordinate Factors

#### Principal Component Analysis of Scales

We computed a principal components analysis (PCA) with Varimax rotation on all scales from the three tests. Both Kaiser criterion and scree plot indicated a three-factor solution that cumulatively accounted for 58.95% of the total variance. Based on their highest factor loadings, MPQ Cultural Empathy, MPQ Openmindedness, CQS Behavioral, CQS Motivational, CQS Metacognitive, ICAPS Flexibility, and ICAPS Critical Thinking loaded on the first component. ICAPS Emotional Robustness, MPQ Emotional Stability, MPQ Flexibility, and MPQ Social Initiative loaded on the second component. CQS Cognitive and ICAPS Openness (negatively) loaded on the third component.

We computed three component scores by averaging the variables above (ICAPS Openness was reversed on the third component) and then computed a simultaneous multiple regression predicting SWLS using the three component scores. The overall model was significant (Table 3). Notably, the effect size was not appreciably different than those using the tests separately. The first component did not contribute uniquely to the prediction, but the second and third components did, with the largest contribution coming from the second component. RWA analyses indicated that only the second component significantly contributed to the prediction of SWLS, accounting for almost 84% of the prediction; there were no differences in these analyses between the United States and Chinese samples. Thus, combining scale scores of the three tests did not appear to account for adaptation more than any of the tests singly.

#### Exploratory Factor Analysis of Individual Items

Using a different method to search for superordinate factors, we computed an exploratory factor analysis (EFA) on all MPQ, CQS, and ICAPS items after standardizing each item within each country/language group. Kaiser criterion indicated the existence of 29 factors that cumulatively accounted for 58.49% of the total variance. Although the variance accounted for was comparable to that in the PCA, a 29-factor solution did not represent adequate data reduction. The scree plot indicated a five-factor solution that accounted for 28.67% of the total variance (see Supplementary Material for additional analyses involving parallel analysis to uncover the number of factors to extract).

Table 4 shows the top ten items from the Varimax rotated component matrix of the five-factor solution, along with notations for the original tests and scales from which each item came. We computed five new scales by averaging all items based on their highest factor loadings using a criterion of >0.30 and labeled them Assertiveness, Attentiveness to Others, Emotional Robustness, Caution (items loading on this scale were negatively scored on ICAPS OP and MPQ CE), and Self-Awareness. Reliabilities were all high (0.80 < α < 0.94). A simultaneous multiple regression on SWLS using the five new scale scores as predictors produced significant results, with independent contributions by Assertiveness, Attentiveness to Others, Emotional Robustness, and Self-Awareness (Table 3). The *R* was comparable to that using the combined original scales of the MPQ, CQS, and ICAPS and was higher than that when using any of the three tests separately, but this analysis suggested a different set of psychological skills underlay the scores.

**TABLE 4:** Rotated factor matrix of the five factor solution from the EFA, top ten items each factor.

Original test	Original scale	Assertiveness	Attentiveness to others	Emotional robustness	Caution	Self-awareness
Alphas		0.944	0.876	0.888	0.801	0.871
MPQ	SI	0.639	0.139	–0.115		
MPQ	SI	0.623		–0.131		
CQS	MO	0.619	–0.154			0.221
CQS	CO	0.593	–0.153	–0.122	0.232	0.183
CQS	MO	0.587				0.391
MPQ	FL	0.579				
MPQ	SI	0.556	0.166	0.140		
MPQ	FL	0.555	0.170			
MPQ	OP	0.554				0.151
MPQ	OP	0.550	0.135	0.109	–0.133	0.121
MPQ	CE	0.136	0.492		–0.197	
ICAPS	CT		0.485		–0.291	0.180
MPQ	CE	0.212	0.478	0.136	–0.257	0.202
MPQ	CE	0.172	0.452		–0.119	0.247
ICAPS	CT		0.443			0.225
ICAPS	CT		0.435			
MPQ	FL	0.104	0.433	0.204		
MPQ	CE	0.205	0.430		–0.169	
MPQ	FL		0.419	0.220		0.189
MPQ	CE	0.320	0.410	0.114	–0.103	
MPQ	ES		0.103	0.730	0.101	
MPQ	ES			0.683		
ICAPS	ER	–0.104	0.201	0.570		0.104
MPQ	ES			0.569	0.182	
ICAPS	ER	0.122		–0.552	0.213	
MPQ	ES			0.541	0.194	
ICAPS	ER	–0.157		0.528	0.258	
ICAPS	ER		0.118	0.527	0.199	
MPQ	ES		0.125	0.519	0.106	
ICAPS	ER	–0.167		0.506	0.161	
ICAPS	OP	0.102			0.520	–0.206
ICAPS	OP			0.116	0.509	
ICAPS	OP		–0.122	0.210	0.481	
ICAPS	OP	0.133			0.467	–0.158
MPQ	CE		–0.202		0.442	
ICAPS	OP				0.441	
ICAPS	OP				0.416	
ICAPS	OP				0.415	–0.144
ICAPS	OP			0.357	0.413	–0.130
ICAPS	OP		0.325	0.110	0.390	
CQS	BE	0.163	0.226			0.776
CQS	BE	0.163	0.203			0.674
CQS	BE	0.253	0.133			0.670
CQS	BE	0.184	0.194			0.667
CQS	BE	0.200	0.165			0.540
CQS	ME	0.242	0.234			0.442
CQS	ME	0.364	0.234			0.440
ICAPS	FL	0.156	0.125			0.316

*MPQ CE, Cultural Empathy; MPQ OP, Openmindedness; MPQ SI, Social Initiative; MPQ ES, Emotional Stability; MPQ FL, Flexibility; CQS ME, Metacognitive; CQS CO, Cognitive; CQS MO, Motivational; CQS BE, Behavioral; ICAPS OAP, Overall Adjustment Potential; ICAPS ER, Emotional Robustness; ICAPS OP, Openness; ICAPS FL, Flexibility; ICAPS CT, Critical Thinking.*

Assertiveness and Attentiveness to Others were both positively associated with SWLS. Emotional Robustness was negatively associated, but inspection of the items loading on this scale indicated that the scale items were worded negatively; thus, the proper interpretation of this finding was that higher emotional robustness was associated with better adaptation. Self-Awareness was also negatively associated with SWLS. Inspection of the items loading on this scale indicated that the scale was essentially comprised of items from CQS Behavioral, which was also negatively associated in the regression including CQS scales (Table 3, top and middle).

RWA indicated that four scales (all but Caution) significantly contributed to the prediction of SWLS, with Emotional Robustness contributing the most (56.88%), followed by Assertiveness (22.15%), Attentiveness to Others (10.96%), and Self-Awareness (7.95%). Once again, there were no differences between the United States and Chinese samples on any RWA finding.

#### Associations Between MPQ, ICAPS, CQS, and New Scale Scores

In order to map how the original scales associated with the five new scale scores from the EFA, we computed correlations between the original scales and the new scale scores (Table 5). Considerable overlap existed between various combinations of the original scales and the five new scales, suggesting that each of the original scales tapped into the five factors in different combinatorial ways. The five scales, however, suggested different conceptual organizations of the constructs underlying the scales.

**TABLE 5:** Correlations between scale scores from the MPQ, CQS, and ICAPS and the five new scale scores.

	Assertiveness	Attentiveness to Others	Emotional Robustness	Caution	Self-Awareness
MPQ CE	0.62**	0.79**	–0.02	−0.21**	0.43**
MPQ OP	0.89**	0.55**	–0.05	–0.01	0.46**
MPQ SI	0.73**	0.28**	−0.33**	−0.20**	0.27**
MPQ ES	0.31**	–0.02	−0.90**	−0.19**	0.12*
MPQ FL	0.16**	−0.42**	−0.33**	−0.33**	−0.11*
CQS ME	0.61**	0.45**	–0.08	–0.01	0.61**
CQS CO	0.64**	0.09	−0.19**	0.12*	0.27**
CQS MO	0.74**	0.31**	−0.16**	–0.08	0.54**
CQS BE	0.44**	0.43**	0.02	–0.05	0.95**
ICAPS OAP	0.42**	0.25**	−0.58**	−0.43**	0.28**
ICAPS ER	0.30**	–0.01	−0.88**	−0.32**	0.02
ICAPS OP	–0.09	–0.01	−0.28**	−0.91**	0.00
ICAPS FL	0.50**	0.44**	0.03	–0.04	0.40**
ICAPS CT	0.30**	0.62**	0.15**	0.32**	0.35**

*p < 0.05, **p < 0.01. MPQ CE, Cultural Empathy; MPQ OP, Openmindedness; MPQ SI, Social Initiative; MPQ ES, Emotional Stability; MPQ FL, Flexibility; CQS ME, Metacognitive; CQS CO, Cognitive; CQS MO, Motivational; CQS BE, Behavioral; ICAPS OAP, Overall Adjustment Potential; ICAPS ER, Emotional Robustness; ICAPS OP, Openness; ICAPS FL, Flexibility; ICAPS CT, Critical Thinking.

We also computed canonical correlations between each of the three original tests and the five new scale scores. Each was highly correlated, *CC* = 0.96, *λ* = 0.003, *F*(25, 1234.83) = 178.66, *p* < 0.001; *CC* = 0.99, *λ* = 0.009, *F*(20, 1105.39) = 170.18, *p* < 0.001; and *CC* = 0.93, *λ* = 0.01, *F*(25, 1227.40) = 111.16, *p* < 0.001, for MPQ, CQS, and ICAPS, respectively.

## Discussion

As predicted, the three tests were intercorrelated and a merged analysis produced superordinate scales that suggested the existence of five different latent constructs. The original scales from the three tests were correlated with the new constructs diversely, with multiple original scales correlated with two to five of the new constructs. Each test predicted SWLS well on its own, as previously documented, but a combination of their scales predicted SWLS better than scales from any test individually. Although superordinate scales using PCA on scale scores did not improve predictions of SWLS, the five new scales did.

The emergence of different latent constructs suggested a novel conceptual framework for understanding the psychological ingredients underlying intercultural adaptation. Assertiveness included items from all five MPQ scales and CQS Motivation, Cognition, and Metacognitive. The items suggested that a psychological competence related to adaptation is a willingness and motivation to engage with the world and put oneself out, to be expressive, self-assured, and confident without aggressiveness. It appeared to be a combination of initiative, knowledge, motivation, and confidence. Theoretically, this construct made sense in that adapting well across cultures and in intercultural interactions may involve an interest to engage proactively with differences and diversity, with the confidence to adapt and adjust effectively. This construct was new to the intercultural adaptation literature and emerged as the first order factor, suggesting its ability to organize items across multiple tests.

Attentiveness to Others was comprised of items mainly from MPQ Cultural Empathy and Flexibility and ICAPS Critical Thinking. This construct suggested that an important psychological ingredient to adaptation is an interest in and paying attention to other people, listening and observing, and doing so within the constraints of cultural rules, norms, and etiquette. Attentiveness to Others also suggested that successful intercultural adaptation requires one not be solely tuned to oneself but also genuinely interested in viewpoints, perspectives, and thinking of diverse others. Although conceptually overlapping with MPQ Cultural Empathy, the addition of items from ICAPS Critical Thinking gave this construct a slightly different twist and thus was somewhat new to the intercultural adaptation literature.

Emotional Robustness was comprised of items from MPQ Emotional Stability and ICAPS Emotional Robustness. This construct has been discussed extensively in the intercultural adaptation (van der Zee and Van Oudenhoven, 2000, 2001; Matsumoto et al., 2001, 2003, 2004) and business literatures (Ayoko and Hartel, 2002; Ayoko et al., 2004). Emotional robustness appears to be important in that engaging with culturally different others and/or diverse environments is replete with emotionally evocative events. Constructively adapting to such events depends on the ability to manage one’s emotional reactions to channel them for constructive ends. Previously, we noted that this is a “gatekeeper” skill of intercultural adaptation because it allows people to access knowledge stores in order to think more critically about how to respond, not react, more adaptively during or after emotionally evocative situations, which are inevitable (Matsumoto et al., 2001). Writers in the intercultural business domain have also commented on the importance of skills related to emotional competence and conflict resolution (Ayoko and Hartel, 2002; Ayoko et al., 2004).

Caution was comprised mainly of items that loaded negatively on ICAPS Openness and MPQ Cultural Empathy. It reflected beliefs concerning a degree of caution or concern about others. It suggested that a psychological competence related to intercultural adaptation may reflect certain degrees of not getting into other peoples’ business or to try to change others or a system (while at the same time being attentive to and empathic with others, as suggested by the other factors). This may be related to the notion that adapting oneself to one’s environment is often easier and more practical than having a culture or system adapt to one.

Self-Awareness was mainly comprised of items from CQS Behavioral and Metacognitive and was negatively associated with adaptation, suggesting that greater self-knowledge was associated with less adaptation. These findings were counterintuitive as self-awareness about one’s own interactive styles should have been positively associated with positive adaptation. The zero-order correlation between CQS Behavioral and SWLS was also negative, *r*(335) = −0.13, *p* = 0.014, and has been reported elsewhere (Rockstuhl and Van Dyne, 2018). This finding should be followed in the future.

All interpretations involving the superordinate structure produced by the factor analysis of all three tests need to be tempered by the small participant-to-item ratio in the analysis, which was a major limitation. Low ratios increase probability of errors, lower accuracies of population estimates, extraction of erroneous factors, and misassignment of items to factors, decreasing external validity and replicability of the solutions (Byrne, 1994; MacCallum et al., 1999, 2001; Tabachnik and Fidell, 2001). The relatively low cumulative variance accounted for along with the high number of factors extracted using Kaiser criterion added to these concerns. To be sure, there is a possibility that the nature of the psychological correlates of intercultural adaptation is such that large pools of items associated with intercultural adaptation produce diverse, multi-structure solutions with many extracted factors using Kaiser and low cumulative variances according to scree, which is what has been reported previously using ICAPS (Matsumoto et al., 2001) and MPQ (Leone et al., 2003; van der Zee et al., 2003; Ponterotto et al., 2007), and again in the current study. However, it is also very possible that the structure reported here is limited to this sample and sample size and should be replicated in the future.

Should the conceptual framework for understanding psychological ingredients underlying intercultural adaptation reported here be replicated, it would have several implications. Theoretically, it would suggest a different conceptualization of the necessary psychological skills and competencies underlying intercultural adaptation in a culture-general framework than previously considered. Although constructs such as emotional stability/robustness/regulation, flexibility, openness, and cultural awareness have often been discussed previously (Ayoko et al., 2004), constructs such as assertiveness, attentiveness to others, or caution are relatively new (although similar constructs have been assessed by other tests reviewed by Matsumoto and Hwang (2013), and other meta-constructs have been presciently proposed and discussed by Van der Zee and Van Oudenhoven (2014, 2017) and Van der Zee et al. (2016). The current findings suggest that the three tests may have predicted intercultural adaptation because of their associations with different latent constructs.

Empirically, a different structure would suggest that future research consider searching for ways of assessing those constructs, and to test boundaries of their utility in accounting for intercultural adaptation. The test and measurement literature is replete with other tests that assess these and similar constructs, especially personality inventories such as the California Psychological Inventory (Gough, 1986), the Minnesota Multiphasic Personality Inventory (Greene, 2000), or the NEO-Personality Inventory-Revised (Costa and McCrae, 2008). There are also many existing tests of constructs related to those produced above, such as self- or emotion regulation (Gross and Levenson, 1993; Carey et al., 2004; Gratz and Roemer, 2004), perspective taking (Long, 1990; Long and Andrews, 1990), assertiveness (Rathus, 1973) or self-efficacy (Bandura, 2005). Future research may engage with these existing methodologies to examine whether a better assessment tool for these, and other, constructs can be produced and better predict intercultural adaptation.

Practically, the identification of a different set of psychological constructs underlying intercultural adaptation would have ramifications for intercultural businesses. Today, international companies are increasingly dealing with risks and demands by sending and receiving workers to and from different countries and cultures, as well as increasingly diverse workforces and customer bases. Identifying an appropriate set of key psychological skills can be important in selecting candidates who would likely fare better and be more effective for overseas or intercultural posts. Knowledge of a core psychological engine would also provide roadmaps for supervisors, mentors, and coaches to guide individuals through critical intercultural incidents and identify areas for self-monitoring and improvement. A conceptual framework for core psychological competencies would also have implications for pre-departure training, in-country coaching and consultation, post-action reviews, and organizational development.

For the intercultural training industry, knowing what kinds of psychological competencies are most related to intercultural adaptation offers the training world with psychological targets that would focus training efforts, methodologies, and pedagogies. Culture-general knowledge of a core psychological engine would impact the structure, format, and content of such training, regardless of the cultures and diversity issues involved. Developing assessment inventories of those concepts would provide the training world with valuable pre- and post-training assessment tools that go beyond experience and anecdotal efforts.

At the same time, identifying and leveraging core psychological competencies underlying intercultural adaptation in a culture-general approach does not negate the importance of other knowledge, skills, and competencies, especially culture-specific ones. Basic to advanced language training is almost always a positive factor, as well as culture-specific information concerning norms, etiquette, rituals, and values concerning daily life, the normal course of human interactions, and specific work settings (Kurman and Ronen-Eilon, 2004). Even knowledge of a culture’s gestures has been positively associated with adaptation (Molinsky et al., 2005). Culture-general and culture-specific approaches are complementary to each other, not contradictory.

That the five new constructs predicted adaptation better than the tests individually was not sufficient to suggest that the newly derived constructs were superior to the original tests, and we make no such claims. Each of the existing tests and their underlying scales have clearly defined conceptual derivations, which are important for both theory and application. Clear and precise theoretical frameworks, which all three tests have, are important for building models of behavior change, with implications not only for intercultural adaptation but also for other domains of life.

Many questions concerning the predictive power and parsimony of any new constructs vis-à-vis the original tests still exist, especially because the adaptation measure used in this study reflected general, not intercultural, adaptation and because the samples were not immigrants or sojourners. Some findings, such as the negative associations between MPQ Flexibility and CQS Behavior with SWLS, may have occurred because the participants were in a monocultural setting. The newly derived constructs, therefore, may not predict intercultural adaptation as well as the original three tests because the latter were designed specifically to do so. (This limitation did not affect the merged analyses producing the five new latent constructs, because these analyses did not include SWLS and were not reliant on intercultural samples.) These issues should be followed in the future, especially in more rigorous designs that include non-convenience, intercultural samples and avoid same-source, same-time, same-methodology assessment of predictors and outcome variables to avoid common method variance or careless responding (Meade and Craig, 2012; Podsakoff et al., 2012).

The SWLS was the only adaptation measure used, and findings obtained concerning associations with adaptation were all limited to this measure; different measures may produce different findings. In particular, we are sympathetic to differences between adaptation and adjustment, the former referring to behavior changes made to adapt to an environment and the latter referring to subjective experiences associated with adaptation (Matsumoto and Juang, 2016). This differential view of adaptation and adjustment is similar to previous discussions concerning differences between sociocultural and psychological adaptation (Searle and Ward, 1990; Ward and Kennedy, 1999; Ward and Szabo, 2019). Future research should replicate and extend the current findings with other measures of adaptation.

Our findings were further limited to the groups sampled, especially the relatively small *n*s for the French and Korean samples. The current findings should be replicated with different cultural and language groups and with more robust sampling to allow for more adequate participant-to-item ratios in order to derive more stable and reliable factor solutions.

Finally, the novel constructs reported here accounted for only a small percentage of the cumulative variance of the items (similar to both ICAPS and MPQ). This suggested that there likely are other meaningful constructs related to intercultural adaptation that we did not tap. Future research should consider engaging in more robust tests of combinations of methodologies and assessment tools to produce new insights concerning the psychological skills and competencies underlying intercultural adaptation.

## Data Availability Statement

The datasets generated for this study are available on request to the corresponding author.

## Ethics Statement

The procedures involving human participants were reviewed and approved by Ethical and Independent Review Services. Written informed consent for participation was not required for this study in accordance with national legislation and institutional requirements.

## Author Contributions

DM and HH contributed equally to the design of the study. HH led the data collection efforts, while DM led the data analysis efforts. DM drafted the first draft of the manuscript and both authors contributed equally in editing to final. Both authors contributed to the article and approved the submitted version.

## Conflict of Interest

DM and HH employees of Humintell, a company that sells one of the tests (ICAPS) utilized in the study described in the article.
