# Supplementary material for: Reconsidering the Active Psychological Ingredients Underlying Intercultural Adaptation: Implications for International Business
Source: Front Psychol. 2020 Sep 24;11:529737. doi: 10.3389/fpsyg.2020.529737 (PMC7541697; doi:10.3389/fpsyg.2020.529737)
Supplement: Supplementary file 1 [file Data_Sheet_1.pdf]

Supplemental Material

One statistical issue concerning the novel five-factor structure presented in the paper concerns the decision criteria for the number of factors retained in the EFA, an issue that is amplified by the relatively low participant-to-variable ratio used in those analyses, and by the fact that the Kaiser criterion and scree plot often do not agree with each other (Preacher & MacCallum, 2003). For this reason, we also conducted parallel analysis (Franklin, Gibson, Robertson, Pohlmann, & Fralish, 1995; Hayton, Allen, & Scarpello, 2004; Lim & Jahng, 2019; Wood, Akloubou Gnonhosou, & Bowling, 2015).

Parallel analysis is a statistical technique in which a Principal Components Analysis (PCA) or EFA (in our case) is computed on a completely random data set that contains the same number of variables as in the original data set. Factors (or components) are extracted and eigenvalues associated with those factors are then compared to eigenvalues produced by an equivalent analysis on the original data set. The root at which an eigenvalue from the parallel analysis is greater than the eigenvalue from the original data set marks the number of factors that could be extracted and interpreted. The logic underlying this procedure is that that root marks where the number of factors produced by totally random data is greater than the number of factors produced by the original data set; thus prior factors may be meaningful because they are greater than what would be generated from random data.

We conducted a parallel analysis on the original item-level data (standardized within country) that produced the novel five-factor solution reported in the Results. As seen in Table S1, eigenvalues from the parallel analysis were larger than eigenvalues from the EFA starting on the 27th root. Because 26 factors did not represent a conceptually meaningful data reduction, these results did not suggest a meaningful method for deciding a number of factors to extract.

The results immediately above may have occurred because of the use of standardized data (within-country), which were used in the original analyses presented in the paper). Thus, we conducted a parallel analysis on the original raw, item-level data as well. As seen in Table S2, eigenvalues from the parallel analysis were larger than eigenvalues generated from the EFA on the 24th root. These results also did not suggest a meaningful method for deciding a number of meaningful factors to extract.

Because the parallel analysis findings themselves may have been affected by the relatively low participant-to-variable ratio, we reduced the number of items analyzed by selecting only the top 10 items on each of the five factors and created a new data set with $k = 48$ items (the fifth factor only included eight items in the original results). We then conducted a parallel analysis on this 48-item data set (standardized within-country). Eigenvalues from the parallel analysis did not go above the eigenvalues generated from the new EFA *anywhere* (Table S3). Thus, we interpreted that the failure of parallel analysis above to suggest a meaningful number of factors to extract was not a result of the participant-to-variable ratio.

Inspection of a fixed, five-factor solution on the reduced data set, however, indicated that exactly the same items reported in Table 4 of the article loaded on the same factors, with no items loading on a different factor. To examine further the overlap between the item loadings between the EFA on the reduced data set and the original data set, we computed Tucker's Coefficients of Congruence (Lorenzo-Seva & Berge, 2006) to examine the overlap of the items

on each of the five factors between the original EFA reported in the ms and the one generated here. The Congruence Coefficients were as follows:

- Factor 1: .997
- Factor 2: .993
- Factor 3: .999
- Factor 4: .996
- Factor 5: .995

We concluded from these supplemental analyses that:

1. Parallel analysis was inconclusive in providing a method for interpreting a meaningful number of factors because all analyses using it produced much too many factors to represent a meaningful data reduction. Interestingly, however, the production of a large number of factors in the parallel analyses was consistent with our contention in the paper (and previous findings) that the number of factors that are associated with psychological ingredients related to adjustment is probably very large.
2. The EFA on the reduced data set produced exactly the same factor loading structure, with no items loading on any other factor, and extremely high Coefficients of Congruence. This provided additional evidence for the loading of those items on the factors reported in the paper.

References

Franklin, S. B., Gibson, D. J., Robertson, P. A., Pohlmann, J. T., & Fralish, J. S. (1995). Parallel Analysis: a method for determining significant principal components. *Journal of Vegetation Science, 6*(1), 99-106. doi:10.2307/3236261

Hayton, J. C., Allen, D. G., & Scarpello, V. (2004). Factor Retention Decisions in Exploratory Factor Analysis: a Tutorial on Parallel Analysis. *Organizational Research Methods, 7*(2), 191-205. doi:10.1177/1094428104263675

Lim, S., & Jahng, S. (2019). Determining the number of factors using parallel analysis and its recent variants. *Psychological Methods, 24*(4), 452-467. doi:10.1037/met0000230

Lorenzo-Seva, U., & Berge, J. M. F. t. (2006). Tucker's Congruence Coefficient as a Meaningful Index of Factor Similarity. *Methodology, 2*(2), 57-64. doi:10.1027/1614-2241.2.2.57

Preacher, K. J., & MacCallum, R. C. (2003). Repairing Tom Swift's Electric Factor Analysis Machine. *Understanding Statistics, 2*(1), 13-43. doi:10.1207/S15328031US0201_02

Wood, N. D., Akloubou Gnonhosou, D. C., & Bowling, J. W. (2015). Combining Parallel and Exploratory Factor Analysis in Identifying Relationship Scales in Secondary Data. *Marriage & Family Review, 51*(5), 385-395. doi:10.1080/01494929.2015.1059785

Table S1

Parallel Analysis Results on Original Standardized (Within-Country) Item-level Data

Parallel Analysis Results			EFA Results[a]		
Random Data Eigenvalues			Extraction Sums of Squared Loadings		
Root	Means	Prcntyle	Total	% of Variance	Cumulative %
1	2.295341	2.395484	20.325	12.244	12.244
2	2.200918	2.281651	11.121	6.700	18.944
3	2.126494	2.179687	7.284	4.388	23.331
4	2.072339	2.121255	4.474	2.695	26.027
5	2.015225	2.073234	3.680	2.217	28.243
6	1.961478	2.007313	3.515	2.117	30.361
7	1.915409	1.965044	2.915	1.756	32.117
8	1.876261	1.917283	2.856	1.720	33.837
9	1.832604	1.876088	2.620	1.578	35.415
10	1.794322	1.842289	2.167	1.305	36.721
11	1.758145	1.807005	2.122	1.278	37.999
12	1.724764	1.767111	2.005	1.208	39.207
13	1.690357	1.721207	1.948	1.173	40.380
14	1.659004	1.693127	1.874	1.129	41.509
15	1.625895	1.659631	1.848	1.113	42.622
16	1.592296	1.627026	1.740	1.048	43.670
17	1.561401	1.593502	1.706	1.028	44.698
18	1.534249	1.565289	1.621	0.977	45.675
19	1.504571	1.532502	1.600	0.964	46.638
20	1.474587	1.508446	1.555	0.937	47.575
21	1.447951	1.478735	1.543	0.930	48.505
22	1.421136	1.452003	1.475	0.889	49.394
23	1.393694	1.424754	1.462	0.881	50.274
24	1.368801	1.393687	1.449	0.873	51.147
25	1.342410	1.372134	1.405	0.846	51.994
26	1.316717	1.346522	1.353	0.815	52.809
27	1.294274	1.320722	1.281	0.772	53.580
28	1.271465	1.298122	1.267	0.763	54.344
29	1.243530	1.271132	1.253	0.755	55.098
30	1.220370	1.248406	1.183	0.712	55.811
31	1.197127	1.220860	1.161	0.699	56.510

32	1.173909	1.199640	1.120	0.675	57.185
33	1.150181	1.175751	1.109	0.668	57.853
34	1.127900	1.150808	1.086	0.654	58.507
35	1.105858	1.126149	1.072	0.646	59.153
36	1.084028	1.103481	1.050	0.632	59.785
37	1.065279	1.085498	1.017	0.613	60.398
38	1.044787	1.063597	0.995	0.600	60.998
39	1.023037	1.044417	0.972	0.586	61.583
40	1.002855	1.022254	0.971	0.585	62.168
41	.983400	1.006191	0.928	0.559	62.727
42	.962914	.981285	0.905	0.545	63.273
43	.943604	.965944	0.888	0.535	63.808
44	.924149	.945996	0.866	0.521	64.329
45	.905484	.931481	0.847	0.510	64.839
46	.886495	.910978	0.826	0.497	65.337
47	.869390	.890625	0.815	0.491	65.828
48	.850896	.868966	0.801	0.483	66.310
49	.831364	.850152	0.774	0.466	66.776
50	.813737	.837771	0.748	0.451	67.227
51	.797189	.822482	0.744	0.448	67.675
52	.779499	.801666	0.715	0.431	68.106
53	.762302	.780953	0.708	0.427	68.532
54	.745547	.766919	0.686	0.413	68.946
55	.727426	.747384	0.662	0.399	69.344
56	.711400	.732818	0.640	0.386	69.730
57	.694069	.714150	0.636	0.383	70.113
58	.678236	.700812	0.616	0.371	70.484
59	.662213	.682306	0.595	0.358	70.843
60	.646783	.665681	0.586	0.353	71.196
61	.632185	.653867	0.578	0.348	71.544
62	.615645	.636469	0.565	0.341	71.885
63	.600862	.620275	0.559	0.337	72.222
64	.586353	.605391	0.548	0.330	72.552
65	.570263	.588241	0.542	0.326	72.878
66	.557118	.573794	0.522	0.315	73.193
67	.541692	.562070	0.509	0.306	73.499
68	.526047	.541918	0.498	0.300	73.799
69	.511466	.529716	0.491	0.296	74.095
70	.496145	.515874	0.481	0.290	74.385

71	.482489	.502696	0.473	0.285	74.670
72	.469595	.484559	0.465	0.280	74.950
73	.455778	.474824	0.443	0.267	75.217
74	.442034	.459224	0.435	0.262	75.479
75	.427943	.443914	0.432	0.260	75.739
76	.414935	.431596	0.415	0.250	75.989
77	.401844	.418893	0.403	0.243	76.232
78	.388748	.406906	0.393	0.237	76.469
79	.375641	.390658	0.379	0.228	76.697
80	.362529	.378625	0.357	0.215	76.912
81	.350362	.366377	0.357	0.215	77.127
82	.338165	.357175	0.341	0.205	77.332
83	.324416	.340557	0.338	0.204	77.536
84	.311908	.329484	0.336	0.203	77.738
85	.300493	.314138	0.322	0.194	77.932
86	.287257	.302426	0.317	0.191	78.124
87	.274573	.288090	0.299	0.180	78.304
88	.262338	.277593	0.287	0.173	78.476
89	.250392	.266610	0.283	0.170	78.647
90	.238513	.254275	0.276	0.166	78.813
91	.227116	.239929	0.265	0.160	78.972
92	.216189	.229284	0.260	0.157	79.129
93	.204579	.216794	0.256	0.154	79.283
94	.193629	.206840	0.245	0.147	79.430
95	.182286	.197061	0.235	0.141	79.572
96	.170736	.182294	0.225	0.136	79.707
97	.159915	.171752	0.210	0.126	79.834
98	.149444	.162535	0.207	0.124	79.958
99	.139003	.152821	0.201	0.121	80.080
100	.127918	.140587	0.189	0.114	80.193
101	.116941	.131414			
102	.106125	.119865			
103	.095744	.108765			
104	.085155	.098065			
105	.075009	.087672			
106	.064631	.079499			
107	.054436	.066635			
108	.043770	.057281			
109	.033543	.047678			

110	.024211	.036697
111	.014843	.026791
112	.005947	.018966
113	-.004338	.008734
114	-.013364	-.001978
115	-.022501	-.011499
116	-.031932	-.018153
117	-.041128	-.028676
118	-.050424	-.038157
119	-.059158	-.049773
120	-.068318	-.058450
121	-.076427	-.066728
122	-.085873	-.077039
123	-.094068	-.082529
124	-.102031	-.091863
125	-.110374	-.099718
126	-.119077	-.108351
127	-.127678	-.118768
128	-.136057	-.124793
129	-.144330	-.136638
130	-.152261	-.143010
131	-.160147	-.150390
132	-.168207	-.158848
133	-.176212	-.167822
134	-.184005	-.175391
135	-.191766	-.181841
136	-.199024	-.190328
137	-.206468	-.198471
138	-.213705	-.204917
139	-.220737	-.212404
140	-.228215	-.219308
141	-.235420	-.225933
142	-.243295	-.234337
143	-.249899	-.241448
144	-.257594	-.251242
145	-.264676	-.257930
146	-.271618	-.264798
147	-.277738	-.270466
148	-.284745	-.278489

149	-.291758	-.284881
150	-.298084	-.290246
151	-.304876	-.297659
152	-.311232	-.305146
153	-.318118	-.310756
154	-.324356	-.317064
155	-.330581	-.323880
156	-.336730	-.330318
157	-.343449	-.336483
158	-.349869	-.342144
159	-.356284	-.350340
160	-.363004	-.356825
161	-.369086	-.363333
162	-.375082	-.368850
163	-.381884	-.376158
164	-.389092	-.382570
165	-.395937	-.388430
166	-.405229	-.397758

[a] EFA could not be computed with 166 factors; thus, we extracted 100 factors for comparison with the parallel analysis results.

Table S2

Parallel Analysis Results on Original Item-level Raw Data

Parallel Analysis Results			EFA Results		
Random Data Eigenvalues			Extraction Sums of Squared Loadings		
Root	Means	Prcntyle	Total	% of Variance	Cumulative %
1	2.295341	2.395484	20.142	12.134	12.134
2	2.200918	2.281651	12.003	7.231	19.365
3	2.126494	2.179687	7.474	4.502	23.867
4	2.072339	2.121255	5.230	3.150	27.017
5	2.015225	2.073234	4.135	2.491	29.508
6	1.961478	2.007313	3.505	2.112	31.620
7	1.915409	1.965044	3.101	1.868	33.488
8	1.876261	1.917283	2.795	1.684	35.172
9	1.832604	1.876088	2.723	1.640	36.812
10	1.794322	1.842289	2.518	1.517	38.329
11	1.758145	1.807005	2.198	1.324	39.653
12	1.724764	1.767111	2.098	1.264	40.917
13	1.690357	1.721207	1.953	1.177	42.094
14	1.659004	1.693127	1.882	1.134	43.228
15	1.625895	1.659631	1.879	1.132	44.359
16	1.592296	1.627026	1.763	1.062	45.421
17	1.561401	1.593502	1.717	1.034	46.455
18	1.534249	1.565289	1.678	1.011	47.466
19	1.504571	1.532502	1.562	0.941	48.408
20	1.474587	1.508446	1.529	0.921	49.329
21	1.447951	1.478735	1.487	0.896	50.224
22	1.421136	1.452003	1.480	0.891	51.116
23	1.393694	1.424754	1.453	0.875	51.991
24	1.368801	1.393687	1.361	0.820	52.811
25	1.342410	1.372134	1.353	0.815	53.626
26	1.316717	1.346522	1.315	0.792	54.418

27	1.294274	1.320722	1.249	0.752	55.171
28	1.271465	1.298122	1.216	0.732	55.903
29	1.243530	1.271132	1.194	0.719	56.622
30	1.220370	1.248406	1.180	0.711	57.333
31	1.197127	1.220860	1.159	0.698	58.031
32	1.173909	1.199640	1.096	0.660	58.692
33	1.150181	1.175751	1.081	0.651	59.343
34	1.127900	1.150808	1.064	0.641	59.984
35	1.105858	1.126149	1.041	0.627	60.611
36	1.084028	1.103481	1.012	0.609	61.220
37	1.065279	1.085498	0.966	0.582	61.802
38	1.044787	1.063597	0.955	0.575	62.378
39	1.023037	1.044417	0.942	0.567	62.945
40	1.002855	1.022254	0.929	0.560	63.505
41	.983400	1.006191	0.908	0.547	64.052
42	.962914	.981285	0.895	0.539	64.591
43	.943604	.965944	0.866	0.522	65.113
44	.924149	.945996	0.849	0.512	65.625
45	.905484	.931481	0.810	0.488	66.113
46	.886495	.910978	0.791	0.477	66.589
47	.869390	.890625	0.779	0.469	67.059
48	.850896	.868966	0.753	0.454	67.512
49	.831364	.850152	0.743	0.448	67.960
50	.813737	.837771	0.725	0.437	68.397
51	.797189	.822482	0.706	0.425	68.822
52	.779499	.801666	0.690	0.416	69.237
53	.762302	.780953	0.676	0.407	69.645
54	.745547	.766919	0.674	0.406	70.051
55	.727426	.747384	0.652	0.393	70.444
56	.711400	.732818	0.633	0.381	70.825
57	.694069	.714150	0.623	0.375	71.200
58	.678236	.700812	0.608	0.366	71.567
59	.662213	.682306	0.588	0.354	71.921

60	.646783	.665681	0.574	0.346	72.267
61	.632185	.653867	0.544	0.328	72.595
62	.615645	.636469	0.541	0.326	72.921
63	.600862	.620275	0.533	0.321	73.242
64	.586353	.605391	0.517	0.311	73.553
65	.570263	.588241	0.505	0.304	73.858
66	.557118	.573794	0.493	0.297	74.155
67	.541692	.562070	0.481	0.290	74.444
68	.526047	.541918	0.477	0.287	74.732
69	.511466	.529716	0.454	0.273	75.005
70	.496145	.515874	0.446	0.269	75.274
71	.482489	.502696	0.438	0.264	75.538
72	.469595	.484559	0.432	0.260	75.798
73	.455778	.474824	0.425	0.256	76.054
74	.442034	.459224	0.415	0.250	76.305
75	.427943	.443914	0.409	0.246	76.551
76	.414935	.431596	0.399	0.240	76.791
77	.401844	.418893	0.384	0.231	77.022
78	.388748	.406906	0.381	0.229	77.252
79	.375641	.390658	0.362	0.218	77.469
80	.362529	.378625	0.360	0.217	77.686
81	.350362	.366377	0.353	0.213	77.899
82	.338165	.357175	0.336	0.202	78.101
83	.324416	.340557	0.330	0.199	78.300
84	.311908	.329484	0.311	0.187	78.487
85	.300493	.314138	0.305	0.184	78.671
86	.287257	.302426	0.293	0.176	78.847
87	.274573	.288090	0.285	0.172	79.019
88	.262338	.277593	0.281	0.169	79.188
89	.250392	.266610	0.270	0.163	79.351
90	.238513	.254275	0.262	0.158	79.509
91	.227116	.239929	0.251	0.151	79.660
92	.216189	.229284	0.243	0.146	79.806

93	.204579	.216794	0.238	0.144	79.950
94	.193629	.206840	0.234	0.141	80.091
95	.182286	.197061	0.224	0.135	80.226
96	.170736	.182294	0.208	0.125	80.351
97	.159915	.171752	0.202	0.121	80.472
98	.149444	.162535	0.195	0.117	80.590
99	.139003	.152821	0.190	0.114	80.704
100	.127918	.140587	0.180	0.109	80.813
101	.116941	.131414			
102	.106125	.119865			
103	.095744	.108765			
104	.085155	.098065			
105	.075009	.087672			
106	.064631	.079499			
107	.054436	.066635			
108	.043770	.057281			
109	.033543	.047678			
110	.024211	.036697			
111	.014843	.026791			
112	.005947	.018966			
113	-.004338	.008734			
114	-.013364	-.001978			
115	-.022501	-.011499			
116	-.031932	-.018153			
117	-.041128	-.028676			
118	-.050424	-.038157			
119	-.059158	-.049773			
120	-.068318	-.058450			
121	-.076427	-.066728			
122	-.085873	-.077039			
123	-.094068	-.082529			
124	-.102031	-.091863			
125	-.110374	-.099718			

126	-.119077	-.108351
127	-.127678	-.118768
128	-.136057	-.124793
129	-.144330	-.136638
130	-.152261	-.143010
131	-.160147	-.150390
132	-.168207	-.158848
133	-.176212	-.167822
134	-.184005	-.175391
135	-.191766	-.181841
136	-.199024	-.190328
137	-.206468	-.198471
138	-.213705	-.204917
139	-.220737	-.212404
140	-.228215	-.219308
141	-.235420	-.225933
142	-.243295	-.234337
143	-.249899	-.241448
144	-.257594	-.251242
145	-.264676	-.257930
146	-.271618	-.264798
147	-.277738	-.270466
148	-.284745	-.278489
149	-.291758	-.284881
150	-.298084	-.290246
151	-.304876	-.297659
152	-.311232	-.305146
153	-.318118	-.310756
154	-.324356	-.317064
155	-.330581	-.323880
156	-.336730	-.330318
157	-.343449	-.336483
158	-.349869	-.342144

159	-.356284	-.350340
160	-.363004	-.356825
161	-.369086	-.363333
162	-.375082	-.368850
163	-.381884	-.376158
164	-.389092	-.382570
165	-.395937	-.388430
166	-.405229	-.397758

Table S3

Parallel Analysis Results on Standardized (Within-Country) Item-level Data, 10 Items per Factor (Total 48 Items)

Parallel Analysis Results			EFA Results		
Random Data Eigenvalues			Extraction Sums of Squared Loadings		
Root	Means	Prcntyle	Total	% of Variance	Cumulative %
1	.939819	1.028195	7.085	14.760	14.760
2	.869945	.926716	5.167	10.764	25.523
3	.802734	.858954	3.654	7.613	33.136
4	.750703	.803118	2.476	5.158	38.294
5	.701903	.749491	1.959	4.081	42.375
6	.660156	.709805	1.531	3.190	45.565
7	.616093	.659078	1.314	2.738	48.303
8	.576109	.623012	1.224	2.550	50.853
9	.535514	.580698	1.084	2.259	53.112
10	.497287	.535134	1.059	2.207	55.319
11	.465280	.496127	1.018	2.122	57.441
12	.429423	.458135	0.994	2.070	59.511
13	.398245	.425477	0.960	1.999	61.510
14	.369383	.399482	0.912	1.900	63.410
15	.338831	.372163	0.898	1.870	65.280
16	.305058	.331538	0.886	1.846	67.126
17	.278603	.311902	0.857	1.785	68.911
18	.253995	.285864	0.839	1.749	70.660
19	.224590	.252713	0.781	1.627	72.288
20	.199075	.227599	0.753	1.569	73.856
21	.172825	.198051	0.718	1.495	75.352
22	.149618	.173570	0.698	1.455	76.806
23	.124477	.147099	0.690	1.437	78.243
24	.100545	.124924	0.673	1.403	79.646
25	.075850	.095604	0.642	1.337	80.982

26	.051634	.073055	0.614	1.280	82.262
27	.031201	.048884	0.590	1.229	83.491
28	.008281	.025668	0.561	1.168	84.659
29	-.015492	.005075	0.527	1.098	85.757
30	-.039415	-.020535	0.514	1.071	86.828
31	-.059551	-.036692	0.490	1.021	87.849
32	-.080990	-.058715	0.467	0.973	88.822
33	-.101016	-.073619	0.455	0.949	89.770
34	-.121945	-.105282	0.445	0.927	90.697
35	-.143251	-.126544	0.435	0.906	91.603
36	-.166582	-.147403	0.417	0.868	92.471
37	-.185502	-.168901	0.401	0.836	93.307
38	-.206312	-.188121	0.379	0.789	94.096
39	-.228800	-.209650	0.357	0.744	94.840
40	-.247715	-.230545	0.345	0.720	95.560
41	-.269182	-.251751	0.324	0.675	96.235
42	-.288880	-.271194	0.313	0.652	96.887
43	-.310198	-.289415	0.304	0.634	97.521
44	-.331475	-.314720	0.267	0.557	98.078
45	-.352979	-.333421	0.264	0.550	98.628
46	-.374535	-.350500	0.247	0.515	99.143
47	-.399820	-.381482	0.223	0.465	99.608
48	-.429379	-.402217	0.188	0.392	100.000
